# CO_2_/CH_4_ and H_2_/CH_4_ Gas Separation Performance of CTA-TNT@CNT Hybrid Mixed Matrix Membranes

**DOI:** 10.3390/membranes11110862

**Published:** 2021-11-09

**Authors:** Chhabilal Regmi, Saeed Ashtiani, Zdeněk Hrdlička, Karel Friess

**Affiliations:** 1 Department of Physical Chemistry, University of Chemistry and Technology, Technická 5, 16628 Prague 6, Czech Republic; jamalias@vscht.cz; 2 Department of Polymers, University of Chemistry and Technology, Technická 5, 16628 Prague 6, Czech Republic; hrdlickz@vscht.cz

**Keywords:** cellulose triacetate polymer, TNT@CNT hybrid fillers, mixed matrix membranes, CO_2_ and CH_4_ separation

## Abstract

This study explored the underlying synergy between titanium dioxide nanotube (TNT) and carbon nanotube (CNT) hybrid fillers in cellulose triacetate (CTA)-based mixed matrix membranes (MMMs) for natural gas purification. The CNT@TNT hybrid nanofillers were blended with CTA polymer and cast as a thin film by a facile casting technique, after which they were used for single gas separation. The hybrid filler-based membrane depicted a higher CO_2_ uptake affinity than the single filler (CNT/TNT)-based membrane. The gas separation results indicate that the hybrid fillers (TNT@CNT) are strongly selective for CO_2_ over CH_4_ and H_2_ over CH_4_. The increment in the CO_2_/CH_4_ and H_2_/CH_4_ selectivities compared to the pristine CTA membrane was 42.98 from 25.08 and 48.43 from 36.58, respectively. Similarly, the CO_2_ and H_2_ permeability of the CTA-TNT@CNT membrane increased by six- and five-fold, respectively, compared to the pristine CTA membrane. Such significant improvements in CO_2_/CH_4_ and H_2_/CH_4_ separation performance and thermal and mechanical properties suggest a feasible and practical approach for potential biogas upgrading and natural gas purification.

## 1. Introduction

Natural gas is considered clean energy compared to other fossil fuels [1]. Carbon dioxide (CO_2_), which is a common contaminant of natural gas, has to be removed to a level of <8% to minimize corrosion of the transmission pipeline [2]. Additionally, CO_2_ lowers the calorific value of natural gas, increases transportation costs, and causes atmospheric pollution [3]. Owing to the generation of high-purity natural gas, conventional separation techniques such as pressure swing adsorption (PSA) [4] or cryogenic distillation [5] have been used to date, but additional compression work is required to maintain both high-pressure PSA operation and low-temperature cryogenic distillation processes. These techniques thus incur substantial energy penalties. An ever-growing interest in removing CO_2_ from natural gas has thus accelerated the search for superior technology [6].

Nowadays, membranes, as a state-of-the-art technology, are used for various types of gas (synthesis, natural, or bio)-based separation processes, as a result of their low cost, compact structures, environmental friendliness, low energy consumption, and user convenience, being among the important reasons for consideration [7]. Gas separation using polymeric membranes has achieved important success in high separation performance since the first commercial-scale membrane gas separation system, produced in the late 1970s [8]. Purifying natural gas using such a polymeric membrane is highly beneficial because of its inherent advantages, such as simplicity in operation, lower capital cost, and high energy efficiency, compared to conventional separation technologies [9]. Although polymers are the practical and economical materials for membrane fabrication and thus separate gases based on their different solubilities and selectivities, the separation mechanism is always restrained by the selectivity permeability trade-off boundary [10]. Despite the easy scale-up, modular state, and flexibility of polymeric materials, they also pose lower permeability and selectivity for gas pairs. One of the methods that can facilitate this trade-off issue is using MMMs that contain an inorganic phase in a polymer matrix. MMMs containing a polymer membrane with dispersed inorganic fillers combine the distinctive properties of both the polymer and inorganic materials, i.e., the polymer’s processibility with the molecular sieving effect of the fillers, which thus exhibit higher permeability along with the selectivity of organic–inorganic mixed phases [11,12]. In this context, membrane researchers focus on synthesizing or modifying advanced polymers to elevate both the gas selectivity and permeability of polymeric membranes by incorporating porous/nonporous micro- or nano-sized fillers into the polymer matrix [13]. The addition of such porous/nonporous fillers into the polymer matrix combines the processability of polymeric membranes with good gas separation performance of inorganic membranes, synergistically contributing to the enhancement in membrane separation performance, thus minimizing the trade-off limit of pristine polymeric membranes [14]. These nanoscale fillers possess highly absorptive properties and are selective in nature due to their large specific surface area and abundant active sites. In conjunction with the polymeric membrane network, they can enhance the overall separation performance of the membrane. Despite their great potential, the fabrication of MMMs is still facing the challenges of incompatibility between the fillers and polymer matrix, leading to inhomogeneous filler dispersion and interfacial defects, which ultimately results in the loss of selectivity and mechanical integrity [15,16]. Thus, the major limitation in such MMM preparation is difficulty achieving homogeneous filler dispersion in the continuous polymer phase. It is therefore critical to control and optimize the interfacial interactions for defect-free composite membranes enabling the synergistic effects of both the fillers and polymer phase via proper selection and matching of filler and polymer combinations that have intrinsically good gas transport properties, as well as strong affinity between the two phases [17]. Conventional supersonic treatment is usually inadequate for achieving satisfactory dispersion, and consequently, new filler dispersion techniques have become increasingly crucial for acquiring high-quality MMMs. Creating nanoscale morphologies on the surface of fillers enhances the interfacial adhesion at the nanometer scale, thus reducing the interfacial rigidification [18]. However, this strategy is limited to zeolites and other silicate fillers only. Similarly, functionalization of existing fillers is often carried out to improve their compatibility and dispersion properties in the polymer matrix, thereby creating defect-free interfacial morphology, reducing leaky interfacial voids and loss of selectivity [19,20]. However, this technique is laborious and could even disrupt the structure and lose the intrinsic properties of the native fillers [12]. Thus, much research into potential new materials for gas separation membranes is currently underway. The simultaneous addition of two types of fillers fosters dispersion and disaggregation inside the polymer matrix due to their different surface chemistries [21]. Using fillers of different natures (surface chemistry, morphology, and properties) in the same MMM is believed to produce synergy effects, leading to membranes with better permeation properties than those corresponding to MMMs with only one filler type.

Moreover, porous fillers are not bound by this trade-off, as they can separate gases through physio-sorption and/or size exclusion. Thus, they can achieve both high selectivity and permeability. Using porous micro- or nano-sized fillers in a polymer to improve its permselectivity was first used in the early 1990s for gas and vapor separation [22,23,24]. Zeolites [24,25], carbon nanotubes (CNT) [26], silica [27,28], fullerenes [29], and metal–organic frameworks (MOFs) [30,31] are commonly incorporated or dispersed into the polymeric matrix to form MMMs. MOFs attract much attention as porous fillers due to their high porosity, adjustable pore size, and rich chemical functionality. However, their hybrid nature, possessing organic linker and inorganic nodes, prevents good compatibility with the polymer matrix, leading to polymer–filler interfacial defects, especially when the particles’ sizes are relatively larger than 100 nm [32]. Therefore, it is of great importance to find an effective strategy to overcome this challenge for the fabrication of high-performance membranes for large-scale gas separation applications.

Among others, single-/multi-walled CNT, which show excellent selectivity for condensable penetrates, e.g., CO_2_ and liquids, are the most researched nanotube fillers in the gas separation process [33]. These fillers have received significant attention for developing MMMs for membrane-based separation technologies. The nanoscale dimension of CNT provides a high aspect ratio (>1000), high surface area, frictionless surface, and high surface energy that can interact with most of the gas molecules [34]. The gas transport inside CNT is extremely fast, which is orders of magnitude higher than commonly used porous materials such as zeolite [35]. The rapid transport behavior of small molecules through these tubes can be attributed to the inherent smoothness of their interior channels [36]. Functionalization of CNT by attachment of suitable functional groups such as carboxyl, hydroxyl, or amine onto their conjugated sp^2^ carbon scaffold prevents CNT from agglomeration, favoring dispersion into the polymer matrix [37]. Henceforth, dopamine has been used to functionalize CNT. Many studies have confirmed that CNT as a filler in different polymers has enhanced the gas separation characteristics. This can be attributed to unique separation properties such as simple functionalization and good dispersion in the polymer matrix, improving mechanical strength even with small filler contents, and good control of pore dimension at the nanometer scale [38,39]. Based on their high adsorption affinity and mechanical and thermal properties, CNT nanocomposite membranes containing CNT and polymer are believed to exhibit co-operative and synergic effects between the polymer and carbon phases for gas separation [26,40]. Molecular dynamics simulation has also predicted that diffusion of light gases such as H_2_, CO_2_, and CH_4_ through CNT is faster compared to other materials because of their smooth internal surface [35]. Murali et al. investigated the incorporation of multi-walled carbon nanotubes (MWCNT) on the gas permeation properties of O_2_, H_2_, N_2_, and CO_2_ gases in Pebax-1657 membranes. They showed that the incorporation of MWCNT caused increases in the free volume of the membranes [41]. Tseng et al. showed the highly improved CO_2_ flux and CO_2_/N_2_ selectivity using MWCNT–polyimide nanocomposites [42]. However, the high energy consumption, expensive fabrication technique, and post-purification steps make CNT costly.

TiO_2_ nanoparticles have gained a lot of interest as potential fillers for the development of MMMs due to their favorable physical properties, stability, low cost, and ease of synthesis [43]. Importantly, they exhibit a very high adsorption capacity and size-selective adsorption for certain gases such as CO_2_ [44]. Compared to silica and MgO nanoparticles, TiO_2_ nanofillers have no potential to fuse naturally. Thus, they disperse individually or in nanoscale aggregates, thereby preventing the formation of non-selective voids in the nanoparticle/polymer matrix interface [45]. Omidkhah et al. investigated the effect of the incorporation of TiO_2_ nanoparticles into MMMs based on Matrimid5218. They revealed that the presence of TiO_2_ nanoparticles increases the gas permeability and CO_2_/CH_4_ selectivity of MMMs due to chain packing distraction [46]. Similarly, Liang et al. reported the improved CO_2_/CH_4_ selectivity for a TiO_2_–PES membrane matrix [47]. Given the promising results of TiO_2_ nanoparticles in gas separation and nanotubes in the advanced oxidation process in water treatment, it was surmised that TNT-embedded membranes generate similar results in various applications. For instance, Imai et al. prepared TNTs in porous alumina membranes and showed their potential in various photocatalytic gas reaction and filtration procedures [48]. Jun et al. synthesized functionalized TNTs and investigated their potential application in fuel cells as an additive to proton exchange membranes [49]. However, to the best of the authors’ knowledge, TNTs have not been applied for gas separation application yet. Furthermore, given the fact that TiO_2_ has a high affinity toward CO_2_, an easy fabrication technique, and is low cost, it was hypothesized that the separation characteristics of CTA-based membranes would be enhanced. Thus, TNTs were chosen as an alternative nanofiller for MMMs preparation in CO_2_ separation application. Moreover, CNTs are excellent electron conductors; they have garnered attention as effective support for a TiO_2_-based composite [50], thus preventing TiO_2_ from aggregating.

Numerous novel polymers have been designed and tailored for CO_2_/CH_4_ membrane separation, aiming to increase the polymer size-sieving ability and thus CO_2_/CH_4_ diffusivity selectivity. When these membranes are exposed to real natural gas streams containing minor gas components such as toluene and hexane as impurities, a significant reduction in performance results, both through competitive sorption and plasticization, thus losing the size-sieving ability and leading to the deteriorated quality of CO_2_/CH_4_ separation performance [51]. There is a growing concern over developing polymeric membranes with a reduced plasticization effect and minimal loss of selectivity. Cellulose acetate (CA) has gained interest, becoming one of the few polymers currently used in commercial gas separations, mainly because of its low cost and environmentally friendly resources, mechanically and chemically stability, and ease of industrial-scale fabricated defect-free thin-skinned asymmetric membranes [52]. The gas transport properties of CA membranes are very sensitive to the degree of acetylation. Puleo et al. reported that CA membranes with a higher degree of acetylation are more CO_2_ permeable but are less selective [53]. Cellulose triacetate (CTA) is a type of cellulose acetate produced by replacing the hydroxyl groups of the cellulose repeat unit with the acetyl group. CTA retains high performance for CO_2_/CH_4_ separation under practical operating conditions with the presence of heavy hydrocarbons, largely due to its high CO_2_/CH_4_ solubility selectivity [54], which may not be affected by plasticization induced by such heavy hydrocarbons [55]. Similarly, Liu et al. reported the plasticization tolerance affinity of CTA hollow fiber membranes on natural gas sweetening under mixed H_2_S and CO_2_ gas and heavy hydrocarbon such as toluene [56]. Moreover, CTA membranes are relatively stable in water at pH 3/7, leading to increased permeability without the loss of selectivity. These membranes are also stable in acidic gases such as SO_2_ [57]. Hence, CTA is considered a potential membrane materials in gas separation processes.

The development of MMMs using conventional fillers is significant yet inadequate to meet the increasing expectations for practical applications on a large scale. The challenges faced by these MMMs have urged extensive research on the use of other promising materials, either single or mixed forms, to help mitigate the existing problems to meet the desired membrane separation performance [12]. The use of hybrid inorganic fillers in the polymer matrix has thus become an increasingly important field of research over recent decades. Using filler particles of different natures in the same MMM may produce synergy effects, leading to membranes with better permeation properties than those corresponding to MMMs with only one filler type. The different surface chemistries of fillers may help the dispersion and disaggregation inside the polymer matrix [21]. Glave et al. combined ordered mesoporous silica MCM-41 and layered titanosilicate JDF-L1 hybrid fillers for 6FDA-based copolyimide MMMs and showed a high level of H_2_ permeability and H_2_/CH_4_ selectivity [58]. Velero et al. combined ordered mesoporous silica with MCM-41 type structure and NH_2_-MIL-53(Al) MOF with polysulfone and polyimide polymer separately. They suggested that the complementary interaction of the two types of particles in one membrane matrix would improve the dispersion of the filler phase and result in superior H_2_/CH_4_ separation performance than those with only one type of filler [59]. Although research works on CNT and TiO_2_ nanoparticles as single fillers in MMMs have been conducted, only a handful of literature works can be found on TiO_2_/CNT hybrid filler-based membranes used for different applications. Shaban et al. [60] synthesized TiO_2_ nanoribbon (TNR)/MWCNT/PES blend membranes by phase inversion and showed the good antifouling nature with high removal of Congo red dye (50 mg/L). Feng et al. [61] prepared a TiO_2_-deposited CNT membrane and showed remarkable enhancement in the capacitive deionization performance. Veréb et al. [62] illustrated the intensification of the ultrafiltration of real oil-contaminated water with a pre-ozonated/TiO_2_/CNT nanomaterial-coated membrane. There is still a lack of studies on the performance of TNT and CNT hybrid fillers in the polymer matrix (e.g., CTA) for gas separation. Thus, the main aim of this research was to synthesize and characterize novel MMMs based on TNT@CNT nanofillers dispersed in a CTA polymer matrix and to study the effect of hybrid nanofillers on the gas separation properties of the polymer. In addition, the physicochemical properties, including the morphology, mechanical properties, and thermal stability, were evaluated.

## 2. Materials and Methods

### 2.1. Chemicals

Titanium dioxide (99.9% purity, Degussa), 1-methyl-2-pyrrolidinone (NMP, ACS reagent >99.0%), sulfuric acid (H_2_SO_4_, analytical grade), phosphoric acid (H_3_PO_4_, analytical grade), potassium permanganate (KMnO_4_, >98%), and hydrogen peroxide (H_2_O_2_, analytical grade) were obtained from Sigma-Aldrich. CTA (acetyl content 43–44%) was purchased from Acros Organics. Sodium hydroxide (NaOH, 98.99%) and hydrochloric acid (HCL, ACS reagent >99.0%) were purchased from Duksan Chemical. All of the chemicals were used as received without any further purification.

### 2.2. Synthesis of TiO_2_ Nanotubes (TNT)

The synthesis method of TNT is explained in detail elsewhere [63]. Approximately 2.0 g of P25 Degussa powder was first mixed with 100 mL of 10 M NaOH and stirred for 30 min, followed by microwave hydrothermal treatment (VOS-301SD, EYLA, 195 W power) for 3 h at 150 °C. The mixture was then washed several times with 5 M HCL until complete neutralization, followed by distilled water to remove the associated impurities further. It was then freeze-dried at −83 °C for 48 h.

### 2.3. Modification of CNT

A commercially available CNT obtained from OCSiAl company (Luxembourg, Germany) was further modified in the laboratory using the method described by Ashtiani et al. [64,65]. During the process, 900 mL of 0.1 M H_2_SO_4_ and 120 mL of 0.1 M H_3_PO_4_ were mixed in a beaker and cooled in a refrigerator. Then, 3 g of CNT (single-walled) was mixed into the solution mixture using a Teflon anchor stirrer, followed by adding 0.2 g of KMnO_4_ little by little with constant stirring, maintaining the reaction temperature below 50 °C. The mixture was stirred for 2 h. The obtained suspension was transferred into a mixture of ice and water. H_2_O_2_ was further used to decompose the unreacted KMnO_4_ in the mixture. The final product was mixed with water, maintaining the concentration of 0.1 wt.%, and stirred by an ULTRA-TURRAX^®^ disperser for 1 h at 10,000 rpm. The obtained dispersion was filtered once more, and the final water dispersion product was stored for further use.

### 2.4. Synthesis of the TNT@CNT Matrix

At first, the dopamine-functionalized CTN was prepared using the following procedure: 0.02 g of the CNT was mixed with 50 mL of methanol and sonicated for 1 h; then, 0.04 g of the dopamine–HCL solution was prepared in 20 mL of methanol. The dopamine solution was added to the CNT mixture and stirred for 12 h, followed by hydrothermal treatment using a Teflon line autoclave, maintaining the temperature of 110 °C for 24 h. The mixture was centrifuged and dried in an oven at 60 °C. Functionalization of CNTs via dopamine helps for better interfacial interaction with the TNT and the polymer matrix. Similarly, dopamine also helps improve the dispersibility of CNTs in the polymer matrix.

Second, the TNT@CNT hybrid filler was prepared following the method of Regmi et al. [66] with slight modification. First, 0.1 g of the TNT was mixed with 0.005 g of the functionalized CNT (i.e., 5 wt.% of CNT with respect to TNT) in 100 mL of ethanol, stirred for 2 h, and heated in the autoclave at 110 °C for 24 h. Finally, the mixture was dried in an oven at 60 °C. The hybrid particles were then used as inorganic fillers in the synthesis of the membrane.

### 2.5. Synthesis of Single and Hybrid Filler-Based MMMs

The membrane was prepared following the method described by Regmi et al. [66] with slight modification. The amount of 0.04 g of each nanofiller (CNT, TNT, and CNT@TNT) was mixed with 25 mL of NMP solvent separately in different beakers. The mixtures were sonicated for 30 min, followed by stirring for 4 h. The amount of 1.62 g of the CTA polymer was added to each of the mixtures and stirred overnight in an oil bath at 70 °C to ensure the complete dissolution of the polymer. The mixtures were further sonicated for 15 min and left undisturbed for 4 h. The membranes were then cast in a glass plate using the applicator (Elcometer 3580, Germany). The casted films were left in the air at ambient conditions for the completion of solvent evaporation. The prepared membranes are symbolized as CTA-TNT, CTA-CNT, CTA-TNT@CNT for TNT, CNT and TNT@CNT filler-based CTA membrane and used throughout the text.

### 2.6. Characterizations

The morphology of the prepared samples was determined using scanning electron microscopy (SEM; Tescan LYRA, Brno, Czech Republic, 15 kV accelerating voltage, SE detector) equipped with energy dispersive spectroscopy (EDS; Oxford Aztec, 80 mm^2^, High Wycombe, U.K.) for detailed analysis of element distributions within the materials and/or chemical microanalysis of the present elements. To ensure their excellent conductivity, the samples were placed on a double-sided adhesive tape made of carbon and covered by 2 nm of gold. Similarly, transmission electron microscopy (TEM) analysis of TNT@CNT was performed on a JEM-2200FS (Jeol, Japan) instrument, maintaining the accelerating voltage of 200.00 kV in the TEM imaging mode. The contact angles were measured by Attension Theta Flex Auto 3 (Biolin Scientific, ESPOO, Finland). Measurements were made in the air using the sessile drop cast method. A camera captured images of the drop profile, and a fitting method was implemented to determine the contact angle. The measurements were thermostated by a thermostat Julabo (constant temperature of 25 ± 0.2 °C). X-ray photoelectron spectroscopy (XPS) analysis was carried out using an ESCAProbeP spectrometer (Omicron Nanotechnology, Uppsala, Sweden). Powdered X-ray diffraction (XRD) measurements were performed at a 273.5 K temperature using a Second Generation D2 Phaser X-ray diffractometer (Bruker, Germany) with Cu Kα radiation (λ = 0.15418 nm), an SSD (1D mode) detector, coupled 2θ/θ scan type, and a continuous PSD fast scan mode. The range of measured Bragg angles was from 5 to 80°. The FTIR spectrometer NicoletTM iS50 (Thermo Fischer Scientific, USA) was used to measure the IR spectra of the samples in the range of 500–3600 cm^−1^ (resolution of 1.93 cm^−1^, 200 scans at 1 s per scan). FTIR spectroscopy in the attenuated total reflection (ATR) mode was used to obtain spectra from the membrane pressed against a diamond crystal surface, and finally, the output spectra were obtained in the absorbance mode. The Brunauer–Emmett–Teller (BET) surface area of TNT nanoparticles was determined at the liquid N_2_ (77 K) temperature using the 3Flex (Micromeritics 3Flex, Heidelberg, Germany) adsorption analyzer. Dynamic mechanical analysis (DMA) was carried out using DMA850 (TA Instruments, USA) in the tensile mode to examine the glass transition temperature of the MMMs and their mechanical properties. For each measurement, a sample gauge length of 10 mm and a width of 4 mm was used. A start temperature of 100 °C, soak time of 5 min, end temperature set of 300 °C (measurement auto stop at specimen break), heating temperature rate of 2 °C·min^−1^, force amplitude of 0.02 N, and pre-tension of 0.25 N were used. Moreover, the tensile stress–strain curves were measured using Instron Universal Testing Machine 3365 (Instron, USA) equipped with pneumatic grips, a rubber-coated sample gauge length (initial sample length between the grips) of 10 mm, a sample width of 4 mm (on average), and a crosshead speed of 5 mm min^−1^ until the specimen broke at an ambient temperature. Thermogravimetric analysis (TGA) was performed using the thermal analysis system Setsys Evolution, Setaram (France). All of the TGA experiments of the samples were performed under an N_2_ atmosphere with a flow rate of 60 mLmin^−1^, maintaining the temperature range of 40–800 °C at a rate of 10 °C·min^−1^. The membrane crystallinity and thermal behavior of the MMMs was further evaluated using a differential scanning calorimeter (DSC) technique via Setsys Evolution, Setaram (France). The samples were analyzed over the temperature range from room temperature to 350 °C. A heating rate of 10 °C·min^−1^ was used with the N_2_ atmosphere around the samples.

### 2.7. Gas Sorption and Permeation Measurements

A CO_2_ and CH_4_ sorption experiment was performed gravimetrically at 25 °C in a pressure range from 0.01 to 1.5 MPa using a self-developed sorption apparatus equipped with a calibrated (McBain) quartz spiral balance (sensitivity 15.528 mg/mm) and a charge-coupled device (CCD) camera system detection of the sample–target–point position. A detailed description of the apparatus and the experimental procedure has been described elsewhere [67,68]. The sample was attached to the spiral balance inside the glass tube with string. The glass chamber was evacuated before the measurement to a pressure lower than 10^−3^ mbar by a rotatory oil pump (Trivac D4B, Oerlikon Leybold, Germany). Experiments were performed at 25 °C and a pressure ranging from 0 to 1.5 MPa. After exposure of the sample to the desired gas at a known pressure, the elongation of the quartz spiral was monitored by an automatic optical system until the equilibrium state was reached. Before each gas experiment, a buoyancy test of the metal string and the spiral without the sample was performed under the same pressure. The buoyancy of the sample was then calculated using the following equation [68]:

(1) $$Buoyancy_{sample}=V_{sample}\times \rho_{gas}=m_{sample}\times \frac{\rho_{gas}}{\rho_{sample}}=\frac{m_{sample\;}\times P\times M}{\rho_{sample}\times T\times R}$$

where $V_{sample}$ is the volume of the sample and $\rho_{gas}$ is the density of the gas at a given pressure and temperature, and $\rho_{sample}$ is the density of the sample.

The gas uptake (***m_s_***) was then calculated using the following equation:

(2) $$m_{s}=\left[\frac{m_{sample\;}\times P\times M}{\rho \times k\times T\times R}+P_{eq}\times b-\langle z_{eq}-z_{in}\rangle \right]\times k$$

where $m_{sample\;}$(g) is the mass of membrane sample, ***P*** is the atmospheric pressure, $P_{eq}$ (MPa) is an equilibrium pressure, ***M*** (g mol^−1^) is a molar mass of gas, ρ (g cm^−3^) is a sample density, ***T*** (K) is the thermodynamic temperature, $z_{eq}$ is an average equilibrium position of the spiral, $z_{in}$ is an average initial position of a spiral, ***k*** is spiral constant, ***R*** (J·mol^−1^·K^−1^) universal gas constant, and ***b*** is the buoyancy slope of the suspension system. The buoyancy of the sample was calculated using gas density assuming the ideal gas behavior.

The gas permeation properties of all of the synthesized membranes were determined by single gas as H_2_, CO_2_, and CH_4_ in a custom-built time lag permeation setup. A circular membrane with an active area of 2.01 × 10^−4^ m^2^ was cut and tightly enclosed into a permeation cell for gas permeation measurement. Prior to the sorption and gas permeation experiment, the membranes were kept overnight in a vacuum oven at 50 °C for complete dryness. Similarly, the permeation cell was constantly evacuated with a vacuum pump before each gas experiment to remove its gases. The synthesized membranes were subjected to the test gases, and the data were collected using the SWeTr version 1.13 (2003, Neovision) data acquisition software. All permeation data were obtained when the steady state was reached using the following equation:

(3) $$P=\frac{V}{A\times R\times T}\times \frac{l}{p_{atm}}\times \frac{d_{p}}{d_{t}}$$

where $\frac{d_{p}}{d_{t}}$ is the slope of the pressure increase in the fixed-volume chamber under steady state conditions, ***A*** is the active area of the membrane, ***l*** is the membrane thickness, ***R*** is the universal gas constant, ***T*** is the experimental temperature, and ***P*** is the atmospheric pressure. The permeability measurement was repeated at least three times for each membrane sample, and an average of three values was recorded. The standard deviation was calculated for these experimental values, and the mean deviation was found between 7% and 10% depending on the nature of the membranes and gas. The entire permeation curve was determined, including the initial transient, to determine the diffusion coefficients of the penetrants by the time lag method and the permeability coefficient from the steady state pressure increase rate. The increase in the pressure in the fixed permeate volume was monitored as a function of time as soon as the membrane was exposed to feed gas at a pressure of 1.5 bar. The transient permeation curve, describing the pressure increase at the permeate side after exposure of the membrane to the feed gas, takes the following form [69,70]:

(4) $$P_{t}=P_{0}+{\left(\frac{d_{p}}{d_{t}}\right)}_{0}t+\frac{RTAl}{V_{p}\;V_{m}}p_{f\;}S\left(\frac{D_{t}}{t^{2}\;}-\frac{1}{6}-\frac{2}{\pi^{2}\;}\sum_{1}^{\infty }\frac{{\left(-1\right)}^{n}}{n^{2}}\;exp\left(-\frac{Dn^{2\;}\pi^{2\;}t\;}{t^{2}}\right)\right)$$

where ***P_t_*** is the permeate pressure (bar) at time ***t***(s); ***P*_0_** is the initial pressure, which is usually less than 0.05 mbar; ***(d_p_/d_t_)*_0_** is the baseline slope, which is generally negligible for the defect-free membrane; ***R*** is the universal gas constant (8.314 × 10^−5^ m^3^ barmol^−1^ K^−1^); ***T*** is the absolute temperature (K); ***A*** is the active membrane area (m^2^); ***V_p_*** is the permeate volume (m^3^); ***V_m_*** is the molar volume of a gas at standard temperature and pressure (22.4 × 10^−3^ m^3^ STPmol^−1^ at 0 °C and 1 atm); ***p_f_*** is the feed pressure (bar); ***S*** is the gas solubility [m^3^ STP/(m^3^bar)]; ***D*** is the diffusion coefficient. and ***l*** is the membrane thickness.

Under steady state permeation conditions, the exponential term approaches zero; hence, the equation becomes:

(5) $$P_{t}=P_{0}+{\left(\frac{d_{p}}{d_{t}}\right)}_{0}t+\frac{RTA}{V_{p}V_{m}}\times \frac{p_{f}P}{l}\left(t-\frac{l^{2}}{6D}\right)$$

A plot of ***P_t_*** versus ***t***, after a long time, gives a straight line, which, upon extrapolation, intersects the time axis at $\frac{l^{2}}{6D}$, describing the time lag (Ѳ) in permeation. These equations thus allow for the calculation of the diffusion and permeability coefficient. Assuming the validity of the solution–diffusion model, the solubility coefficient was then determined indirectly by simple relation:

(6) P=S×D

The gas permeability (***P_i_***, 1Barrer = 10^−10^ cm^3^ (STP) cm cm^−2^s^−1^cmHg^−1^) is defined by the given equation:

(7) $$P_{i}=\frac{lQ_{i}}{A\Delta P_{i}}$$

where ***l*** refers to the thickness of the membrane (µm), ***Q*** represents the volume flow rate (cm^3^s^−1^, STP) of gas ***i***, ***A*** is the effective membrane area (cm^2^), and **Δ*P_i_*** is the partial pressure difference across the membrane (cmHg).

The expression that defines the selectivity is:

(8) $$\alpha_{ij}=\frac{P_{i}}{P_{j}}$$

where $P_{i}\;$ and $P_{j}\;$are the permeability of the two pure gases ($P_{i}\;$***>*** $P_{j}$), respectively.

## 3. Results and Discussion

### 3.1. Physico-Chemical Property Evaluation

**Figure 1:**
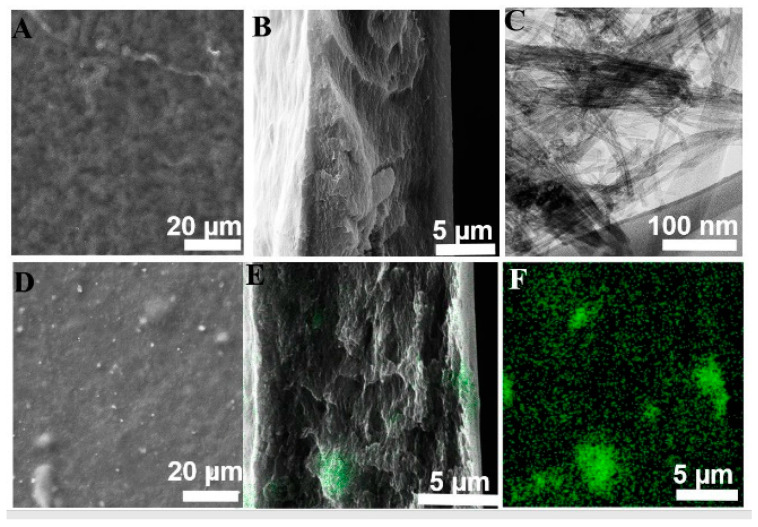
(**A**,**B**) Surface and cross-section of CTA-CNT; (**C**) TEM image of CNT@TNT; (**D**) SEM of the surface view of CTA-TNT@CNT; (**E**) EDS overlayed cross-section image of CTA-TNT@CNT; (**F**) EDS showing Ti distribution of (**E**).

SEM examined the membrane morphologies and polymer–filler interfaces of all membranes. Figure S1 represents the SEM images of the pristine CNT and TNT nanoparticles. The CNT showed a long fibrous structure with varying diameters and being hundreds of nanometers in length, while the rod-like structure of TNT was observed under SEM. The BET surface area of the TNT nanoparticles was measured to be 214.45 m^2^·g^−1^. SEM images (Figure S2A,B) of the TNT@CNT composite illustrate that the CNT was interwoven with TNT particles, which was further evidenced from the interlayer EDS mapping of C and Ti (Figure S2C). TEM images of the pristine TNT and CNT are shown in Figure S3. The TEM image of TNT (Figure S3A) shows that the surface morphology of the samples had an entire nanotube structure. The average diameter of the tubes was approximately 5.06 ± 0.03 nm, and their length was several hundred nanometers. Figure S3B shows the TEM image of oxidized CNT nanoparticles. Long tubular and flexible filament-like particles can be observed. Figure 1 shows SEM and EDS images of the surface, a cross-section of CTA-CNT and CTA-TNT@CNT MMMs, and a TEM image of the CNT@TNT composite. Both CTA-CNT and CTA-TNT@CNT (Figure 1A,D) showed a rough surface with a typical ridged valley structure. The bright spots in the MMM image (Figure 1D) represent the embedded hybrid nanoparticles. These hybrid fillers can be observed to be well dispersed onto the membrane surface. No noticeable gaps or defects can be seen at the single particle–polymer interface.

The TEM image in Figure 1C clearly shows the tube morphology of TiO_2_. Figure 1E shows the EDS overlayed cross-section of the CTA-TNT@CNT membrane. Ti shows an excellent overall dispersion throughout the membrane. The cross-section of Figure 1B,E shows the formation of a highly dense membrane. Mapping Ti (Figure 1F) revealed a homogeneous distribution of Ti throughout the matrix. Only a few clusters or aggregates of the fillers were observed, which is trivial. A homogeneous structure with no interfacial voids between the nanofillers and polymer phase indicates high compatibility between the two phases. The reason for good compatibility is increased interaction between the phases. The measured contact angle for CTA and CTA-TNT@CNT are 53 ± 2° and 60 ± 3° respectively, indicating an increase in the hydrophobicity of the membrane. The images of the liquid droplets on the membrane surface are shown in Figure S4.

**Figure 2:**
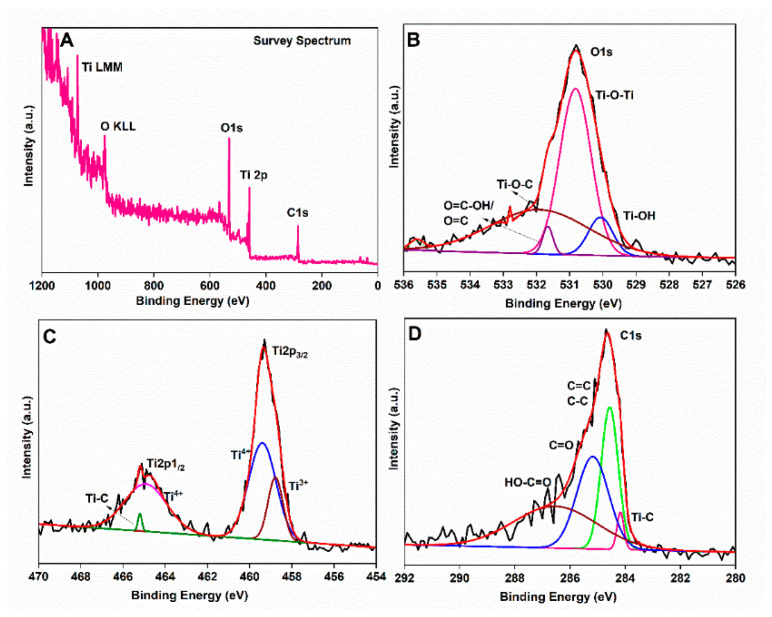
XPS analysis of the CTA-TNT@CNT sample: (**A**) Survey spectrum; (**B**) high-resolution O1s; (**C**) high-resolution Ti2p; and (**D**) high-resolution C1s core level analysis.

Figure 2A shows the survey spectrum of TNT@CNT. The survey scan spectra revealed a peak of the O, Ti, C, and K elements. Two sets of peaks at 458.8 eV and 464.9 eV observed in the Ti2p spectrum can be associated with Ti2p3/2 and Ti2p1/2, which are the characteristic binding energy values for the Ti^+4^ oxidation state. The splitting between 2p_3/2_ and 2p_1/2_ was 5.7 eV. This particular energy gap is an indication of the predominant presence of Ti^4+^ in the system. The peak at 459.9 eV is attributed to Ti^+3^, while the peak at 465.2 is attributed to the Ti–C bond. The high-resolution XPS spectrum for C1s shows different types of carbon bonds. The major peak at 284.5 eV is assigned to C=C, C–C, or C–H bond, while the peaks at 285.2 eV and 286.5 eV are related to the C=O and OH–C=O bonds of carbon-based functional groups, respectively. The peak at 284.1 eV represents Ti–C bond, while the peaks centered at 530.0 eV, 530.9 eV, 531.7 eV, and 531.9 eV in the fitted O1s spectra (Figure 2B) are attributed to Ti–O–Ti, O=C–OH/O=C, and Ti–O–C bonds, respectively [71,72,73]. The existence of Ti–C and Ti–O–C bonds indicate that TNT is substantially bonded to the CNT.

**Figure 3:**
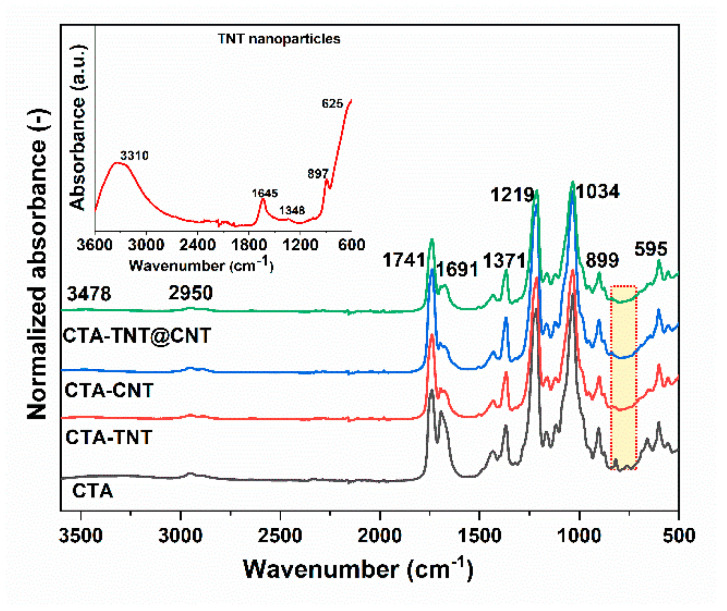
FTIR spectra of the synthesized membrane samples. The inset is the FTIR of the TNT nanoparticles.

Figure 3 shows the FTIR spectra of the pristine and modified membranes. The peak at 3478 cm^−1^ is attributed to the OH stretching vibration in hydroxyl groups, while the band observed at 2950 cm^−1^ is assigned to asymmetric C–H bands in methyl and methylene groups. The absorption band at 1691 cm^−1^ is due to adsorbed water, and the absorption band centered at 1371 cm^−1^ is assigned to the C–H stretch of cellulose and asymmetric deformation of the CH_3_ group for acetates. The bands at 1219 cm^−1^ and 1034 cm^−1^ are attributed to C–O–H and C–O stretching vibration, respectively [74]. The FTIR spectra of the pristine and modified membranes show no significant differences. The disappearance of an absorption peak at 820 cm^−1^, assigned to aromatic –C–H out-of-plane band in the CTA matrix, in MMMs presumed due to the interaction of the polymer matrix with fillers (TNT/CNT) used. The intensity of the characteristic peaks of the CTA matrix in MMMs became weaker. This behavior is attributed to the interaction between the fillers and the polymer matrix, as well as the reduction of the polymer concentration in the sample due to the presence of filler particles [75]. For TNT nanoparticles (Figure 3, inset), the peaks near 625 cm^−1^ and 1346 cm^−1^ are assigned to the lattice vibration of TiO_2_ (Ti–O–Ti stretching). In contrast, OH bending and stretching modes are observed at 1646 cm^−1^, and 3310 cm^−1^, indicating the surface adsorbed OH groups [76].

Similarly, Figure S5 shows the XRD pattern of the fabricated membranes. The broad diffraction peak centered at around 19° in the XRD pattern of pristine CTA confirms the semi-crystalline nature. The TNT nanoparticles show the mixed phase of anatase and rutile. The observable peaks related to both TNT and CNT nanoparticles are not well detected in the MMM samples. This is assumed to be due to the efficient dispersion of the fillers within the polymer matrix. The concentration of the filler at the point of detection is insufficient for the collection of the XRD data.

**Figure 4:**
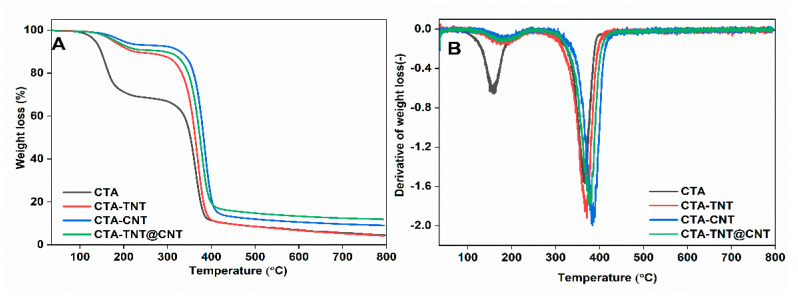
(**A**) TGA and (**B**) corresponding DTG analysis curves of pristine membranes and MMMs in the study.

The effect of inorganic fillers on the thermal behavior of the MMMs was studied by the TGA technique (Figure 4A,B). The pristine membranes and MMMs showed the two stages of thermal degradation in the temperature ranges of 156–182 °C and 370–385 °C. The weight loss in the first phase (156–182 °C) is attributed to loss of the surface moisture held by hydrogen bonding, evaporation of entrapped solvent, and decomposition of a small amount of acetylated cellulose and some esterified chains [74]. Szabo et al. suggested that conformational changes in the side alkyl chains of the polymer and crosslinking causes an elevation in thermal stability [77]. Hence, it is also presumed here that the inorganic fillers used possibly cause crosslinking, which likely reduces the mobility of the CTA chains. This property slows down the degradation process, increasing thermal stability. The second stage of weight loss was attributed to the degradation of the CTA, followed by thermal decomposition. Significant decomposition started at 302 °C, with a major loss at 370.9–384.5 °C for different sample compositions. Comparing the thermal stability between pristine CTA and MMMs, the degradation temperature was around 370.9 °C for pristine membranes, increasing to 384.5 °C for CTA-CNT and 377.7 °C for CTA-TNT@CNT. The degradation temperature was retained within the range of ±14 °C. Furthermore, the highest thermal stability for the CNT-incorporated MMMs is presumed to be due to the excellent thermal conductivity of CNT. The interaction between the polymer chains and the reactive groups on the filler’s surface results in reinforced polymer matrices that restrict the thermal motion of the polymer. This restricted motion increases the energy needed for the movement and segmentation of the polymer chains, thus leading to enhancement of the thermal stability of MMMs [75,78]. Similarly, the high thermal stability of the filler itself adds to the increase in the thermal stability of MMMs compared to pristine membranes. Thus, the improvement in thermal stability is attributed to the strong interaction among the CTA polymer matrix and the TNT/CNT fillers, forming a bonded network structure in the hybrids. This interaction can hinder the mobility of the polymer chains so that the decomposition temperature is increased in MMMs.

**Figure 5:**
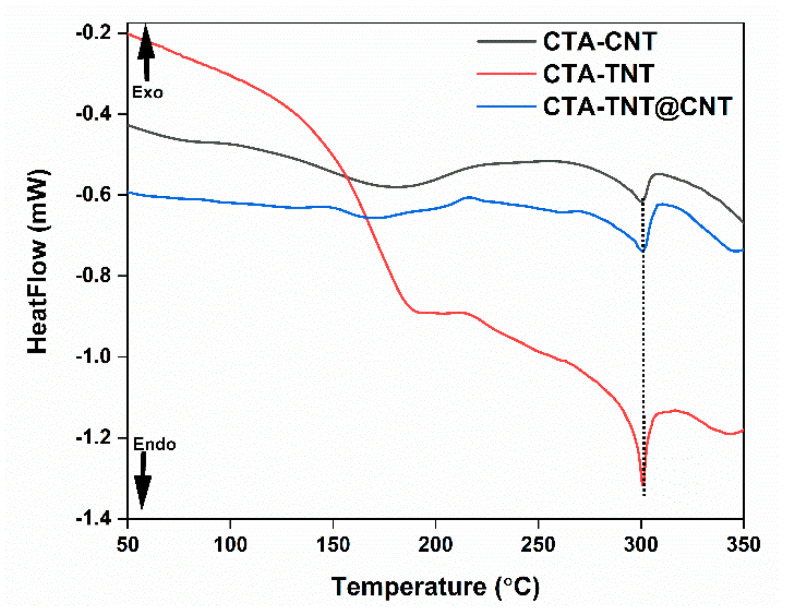
DSC thermograms of the synthesized CTA-TNT@CNT MMMs.

The polymer crystallinity and thermal behavior of the MMMs was evaluated by the DSC technique by quantifying the heat dissociated with fusion of the polymer. This heat is expressed as the percentage of crystallinity by normalizing the observed heat of fusion to the 100% crystalline sample of the same polymer. Figure 5 shows the first heating DSC curves of the synthesized MMMs. The major endothermic peak at approximately 300 °C can be observed for all MMM samples. The DSC spectra, melting temperature, and enthalpy of fusion of the pristine CTA were reported in our previous work [79]. Compared to pristine CTA (melting temperature of 289 °C), incorporating inorganic fillers (TNT/CNT) into the polymer matrix increases the melting temperature to 300 °C. This increase in the melting temperature could be attributed to the good dispersion of the fillers into the polymer matrix [80].

The degree of crystallinity was evaluated from the melting enthalpy in the first scan of each sample. The melting temperature was taken as the peak of the melting endotherm from the first scan of each membrane. The heat of fusion/melting was calculated from the integrated area under the melting peak. The enthalpy of fusion for the pristine CTA membrane was calculated to be 7.31 J·g^−1^ [79], whereas the calculated enthalpy of fusion for CTA-TNT, CTA-CNT, and CTA-TNT@CNT were 13.95 J·g^−1^, 14.41 J·g^−1^, and 19.77 J·g^−1^, respectively. The degree of crystallinity (ℵ) of the modified membranes was calculated by the equation below:

(9) $$ℵ=\frac{\Delta H}{\Delta H^{{}^{\circ}}}\times 100\%$$

where ΔH (J·g^−1^) is the melting enthalpy of the synthesized membrane and $\Delta H^{{}^{\circ}}$ is the melting enthalpy for a 100% crystalline sample of pure CTA. The heat of fusion for a pure CTA crystallite was taken to be 34.31 J·g^−1^ [53], assuming that this heat of fusion applies for all modified membranes. The calculated crystallinity of the MMMs are as follows: 40.65%, 41.99%, and 57.62% for CTA-TNT, CTA-CNT, and CTA-TNT@CNT, respectively. As compared to the degree of crystallinity of the pristine CTA membrane (21.31%) [79], this increased in the degree of crystallinity in MMMs also indicates the successful incorporation of fillers into the polymer matrix [81]. It is generally accepted that the permeability of polymer membranes decreases with an increase in the degree of crystallinity of polymers, as the crystalline zones in polymeric membranes do not contribute to the transport of gas molecules [82]. However, there are few exceptions where the trend is the opposite [83,84,85]. Interestingly, unlike common crystalline polymer membranes, the CNT/TNT-based MMMs with a higher crystallinity value showed larger gas permeability than that of less crystalline pristine CTA membrane (see Section 3.2). Similarly, with the increase in membrane crystallinity, an increase in the CO_2_ diffusion coefficient and no detrimental increase in the solubility coefficient were observed (see Section 3.2). This is opposite behavior compared to the behavior of common crystalline polymer membranes. This indicates that CO_2_ permeability is more dominated by diffusivity than solubility. Besides the contributions of hybrid nanofillers in enhancing permeability and selectivity, additionally, there might be a continuous space, larger than CO_2_ gas molecules, for gas diffusion around the interface between the crystalline and amorphous region, which might be created by the stress of polymer chain arrangements around the interface regions [84], thus promoting the enhancement of permeability. However, further research is essential. Similarly, broad peaks from 150 to 200 °C centered at around 175–190 °C, for different membranes, are associated with the glass transition temperature (T_g_) of the polymer composite. Compared to the T_g_ value obtained from DMA (Table 1), these values obtained from DSC are slightly lower. The different values obtained via different methods is mainly attributed to the frequency effect. Since DSC is sensitive to the heat capacity (C_p_) changes associated with glass transition, while DMA is sensitive to mechanical relaxation, the change in thermal properties on T_g_ (e.g., heat capacity in DSC) is much less than the change in the mechanical behavior in the DMA on T_g_ [86]. Thus, for polymer matrix, the T_g_ results obtained from the DMA technique are considered more precise than those of the DSC method.

**Figure 6:**
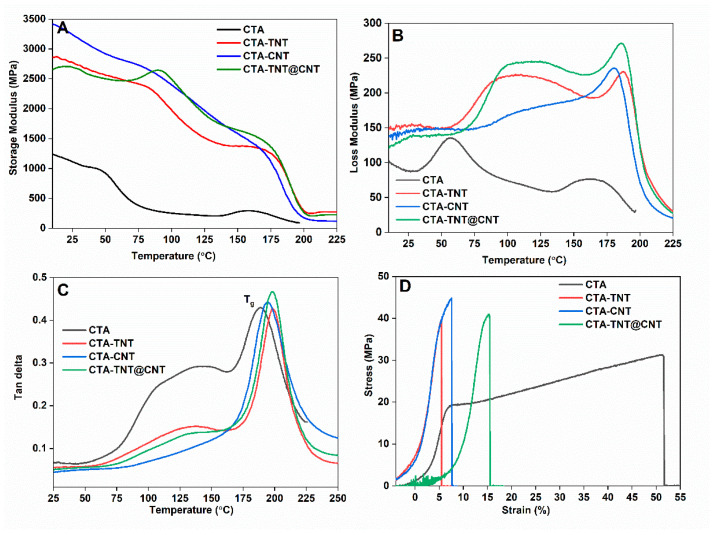
(**A**) Change in storage modulus; (**B**) loss modulus; (**C**) Tan delta as a function of temperature; and (**D**) stress–strain plot for the synthesized membranes.

The changes in the storage modulus, loss modulus and damping factor of pristine membranes and MMMs as a function of temperature determined from DMA testing are shown in Figure 6. The general trend reveals that the magnitude of both the storage and loss moduli increased in the MMMs compared to the pristine CTA, as presented in Figure 6A,B, respectively. Incorporating porous fillers into the CTA matrix increased the energy storage capacity of the composite when subjected to an oscillatory strain; hence, the storage modulus increased. Since the storage modulus is directly proportional to the interface bonding [87], we reasoned that adding the TNT@CNT matrix as a filler may have homogeneous distribution and strong chemical bonds with the polymer matrix, thereby increasing the energy dissipation capacity of the MMMs. Above T_g_, the storage modulus is almost the same and minimum for all the samples, suggesting a great increase in the molecular mobility. Similarly, the loss modulus represents the results of the viscous behavior of the composite, as well as the dissipated energy changed into heat. The maximum values of the loss modulus obtained for the MMMs are higher than those obtained for the pristine CTA. It can also be stated that the mobility of the polymer chain is higher for the mixed matrix, resulting in a bigger plastic effect. The chemical bond between the nanofillers and the polymer matrix is also presumed to be stronger. Similarly, the dampening capacity of the polymer is highly tuned by the addition of the TNT@CNT filler matrix. The higher values of the maximum of the damping factors are due to the possible lower amplitude of the molecular movement of the CTA chain. In MMMs based on CTA-TNT@CNT, the interactions between the polymer chains and nanofiller surfaces were presumed to be more substantial due to the better compatibility and increased bonding with the polymer chains, resulting in higher T_g_ values.

The stress–strain behavior and the values of their corresponding parameters are shown in Figure 6D and Table 1. It can be seen that the tensile strength of the MMMs is higher than that of the pristine CTA, while the elongation at break is lower. The Young’s modulus for the single filler decreased, whereas, for the hybrid filler, it is almost similar to that of the pristine CTA. Generally, an increase in tensile strength indicates good interaction between the organic phase and inorganic filler phase and good dispersion of the fillers throughout the CTA matrix [88]. Similarly, the increase in tensile strength could be because of the development of a co-continuous phase morphology [89]. This suggests the increment of membrane strength, which is presumed to restrict chain segmental mobility by adding inorganic fillers [90]. The decrease in the Young’s modulus in the single filler membrane matrix is attributed to the stress concentration tempted by aggregation of single fillers, whereas for the hybrid fillers, this is due to the increase in polymer chain entanglement with a polar group of CNT and TNT, which leads to constrained polymer chain mobility, resulting in a stronger membrane [91]. The elongation at break of the membrane depends on the ability of the inorganic filler to restrict polymer chain movement and circumvent the occurrence of large macroscale extension [28]. The decrease in elongation at break with the addition of TNT/CNT fillers could be due to the limitation of polymer chain packing movement supported by strong interfacial interaction of polymer chains with fillers. Consequently, the addition of such fillers might prohibit the occurrence of large macroscale expanse and increase the MMMs, resulting in a more rigid but brittle structure of MMMs [92].

### 3.2. Gas Separation Performance Evaluation

**Figure 7:**
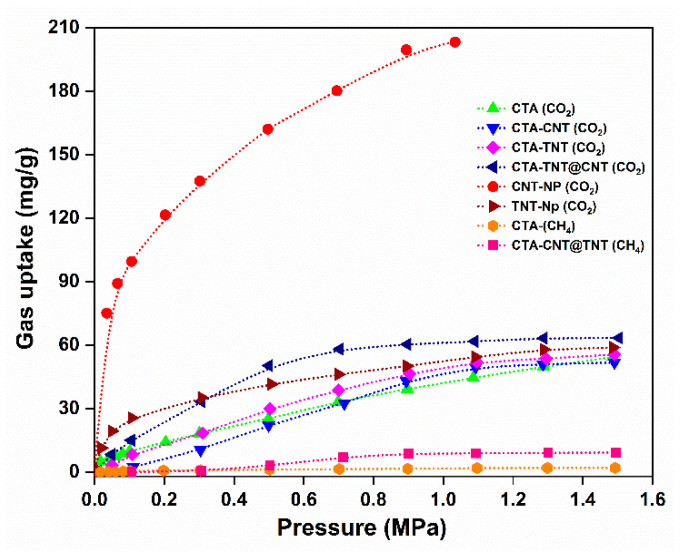
Pure CO_2_ and CH_4_ gas uptake capacity of the synthesized MMMs. The dotted lines are a guide for the eye.

To investigate the role of TNT, CNT, and TNT@CNT fillers, gravimetric adsorption of pure CO_2_ and CH_4_ was performed. The CO_2_ and CH_4_ sorption affinity for all of the synthesized membranes measured at 25 °C and a pressure range of 0.01–1.5 MPa is plotted in Figure 7. The CO_2_ uptake affinity of all the sets of samples was higher than that of CH_4_ uptake, which could be presumed to the high condensability of CO_2_ gas and the more vital interaction between the polar gas (CO_2_) and the MMMs. The CH_4_ uptake efficiency of the CTA-TNT and CTA-CNT-based membranes was very low for the given mass of samples below the device’s sensitivity. The CNT nanoparticles showed the highest CO_2_ sorption affinity among all of the samples in the study owing to their high surface area, tunable pore structure, as well as the presence of the polar groups as useful sites for binding of CO_2_. Notably, the TNT nanoparticles showed higher CO_2_ sorption affinity than the pristine CTA in the low-pressure range up to 0.5 MPa and lower at higher pressures above 0.5 MPa, suggesting earlier saturation behavior. The sorption affinity of the TNT particles is attributed to the –OH group, which possesses a high affinity toward CO_2_. The CO_2_ uptake affinity of the hybrid (TNT@CNT) filler-based MMMs was higher than that of the pristine CTA and single filler (CNT/TNT)-based MMMs. All of the MMMs tended toward saturation at high pressures above 0.8 MPa, whereas the sorption affinity of the pristine membrane increased with an increase in pressure. This is attributed to the saturation of the active sites of fillers with CO_2_ gas with an increase in CO_2_ concentration. The difference in CO_2_ and CH_4_ adsorption capacity confirms the feasibility of enhancing the separation performance.

**Figure 8:**
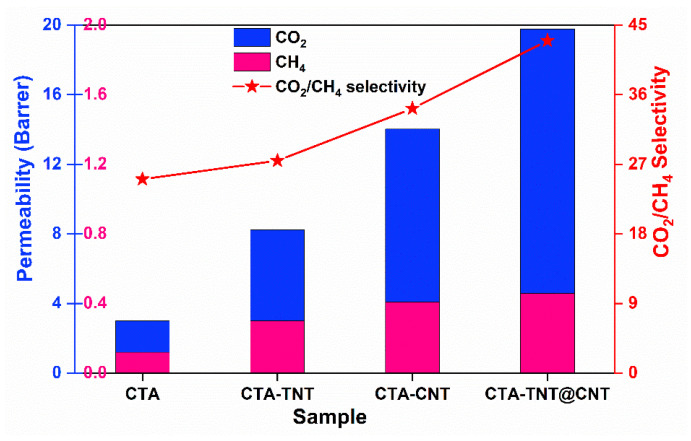
Single gas CO_2_ and CH_4_ permeability and selectivity plot of the synthesized membranes.

The ideal gas separation performance of the pristine membranes and MMMs was investigated by single gas (H_2_, CO_2_, and CH_4_) permeability measurements, and the results are shown in Table 2. The feeding pressure and operating temperatures were 1.5 bar and 25 °C, respectively. The kinetic diameter of the tested gases increased in the order of H_2_ (2.89 Å) < CO_2_ (3.3 Å) < CH_4_ (3.8 Å). The permeability results correlated with the size of gas molecules. It can be seen that the permeability of the gases increased with a decreasing kinetic diameter, resulting in higher permeability for gas with a smaller kinetic diameter. As shown in Figure 8, the pristine membrane showed a CO_2_ permeability of 3.01 Barrer with a CO_2_/CH_4_ selectivity of 25.08. The addition of hybrid fillers (TNT@CNT) led to an almost six-fold increase in permeability. The CO_2_/CH_4_ selectivity increased from the single fillers (TNT/CNT) to the hybrid fillers (TNT@CNT), reaching 42.98 following the order of: CTA < CTA-TNT < CTA-CNT < CTA-TNT@CNT.

Interestingly, the CO_2_ permeability and CO_2_/CH_4_ selectivity were enhanced after incorporating porous fillers. The gas permeability and CO_2_/CH_4_ selectivity were strongly correlated to the single and hybrid filler types, indicating the pivotal role of fillers in MMMs for gas separation. A continuous increase in selectivity was observed, explaining the appearance of a barrier effect upon the addition of hybrid nanofillers. Comparing the gas sorption between CO_2_ and CH_4_, the more absorbable gas molecules (CO_2_) adsorbed on the membrane surface, then moved along to the permeate side on the surface of the pores until desorbing the gas. The highly absorbable gas had higher permeability than the less absorbable gas molecules (CH_4_), with lower permeability. Thus, the selectivity of the MMMs increased compared to the pristine membranes.

**Figure 9:**
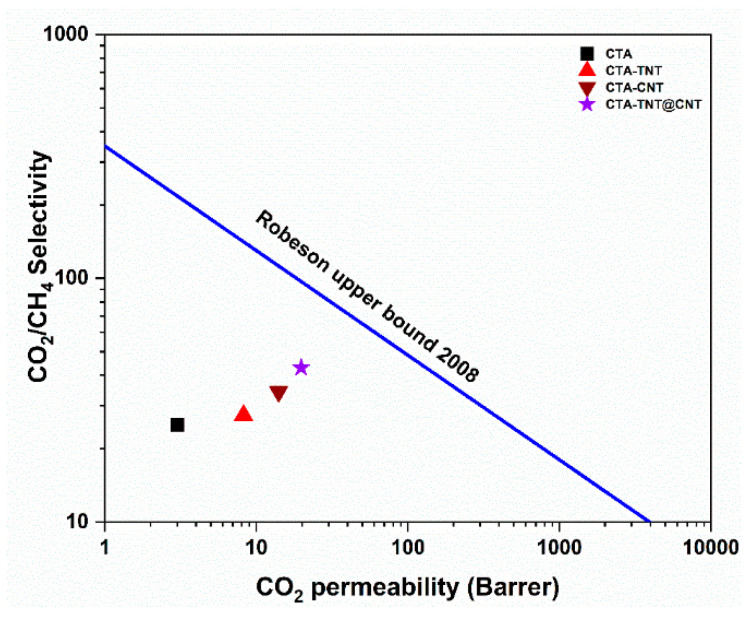
Plot of CO_2_/CH_4_ separation efficiency of the synthesized membranes compared to the Robeson upper-bound plot [93].

The Robeson upper bound plot is extensively adopted to provide visualized comparisons among membrane separation performance. The pure CTA membrane exhibited a CO_2_/CH_4_ separation performance far below the Robeson upper-bound limit, significantly upshifted with incorporating single (CNT, TNT) and hybrid (TNT@CNT) fillers, as shown in Figure 9. The efficiency of separating CO_2_ from CH_4_ was presumed to be the synergistic effect of the porous nature of the hybrid fillers.

**Figure 10:**
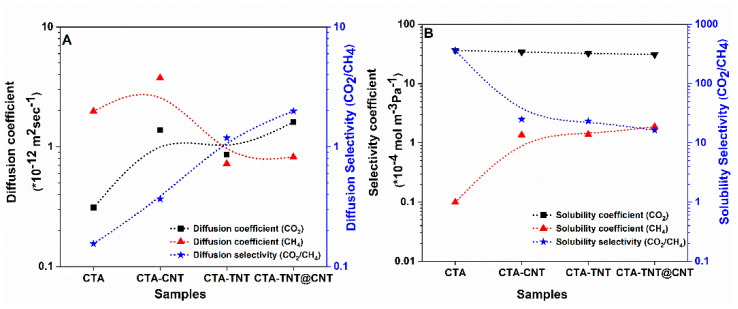
(**A**) Diffusion coefficient of CO_2_ and CH_4_ and their respective selectivities. (**B**) Solubility coefficient of CO_2_ and CH_4_ and the corresponding selectivities of the synthesized samples (dotted lines are a guide for the eye).

To understand the contribution of diffusivity and solubility to the permeability of the gases, the diffusion coefficients, solubility coefficients, and the corresponding selectivities for CO_2_ and CH_4_ were calculated. The results are shown in Figure 10. The solubility of CO_2_ was higher than that of CH_4_ for all of the membranes, which is attributed to its greater condensability and better affinity to the membrane matrixes. No detrimental difference in the solubility of CO_2_ was observed in the MMMs compared to the pristine CTA. On the contrary, the solubility of CH_4_ increased for the MMMs, reaching the highest value for the CTA-TNT@CNT membrane. On the other hand, the diffusion coefficient of CO_2_ increased from the pristine CTA membrane to the CTA-TNT@CNT membrane, while the CH_4_ diffusion coefficient was in the reverse order. Compared to the pristine CTA membrane, the diffusion selectivity gradually increased and reached maximum for the CTA-TNT@CNT membrane, while a slight decrease in the solubility selectivity was observed for the MMMs. However, the decreased value of solubility selectivity was even higher than the highest value of the diffusion selectivity. Thus, the overall increase in the permeability/selectivity in the MMMs resulted from the synergistic effect of the solubility selectivity and diffusion selectivity. The enhanced permeability of the MMMs compared to the pristine CTA membrane is attributed to the increased diffusion coefficient, particularly CO_2_. This increase in CO_2_ permeability is imputed to the rise of gas solubility and diffusivity, while the rise of CO_2_/CH_4_ separation selectivity is mainly ascribed to the higher value of solubility selectivity.

**Figure 11:**
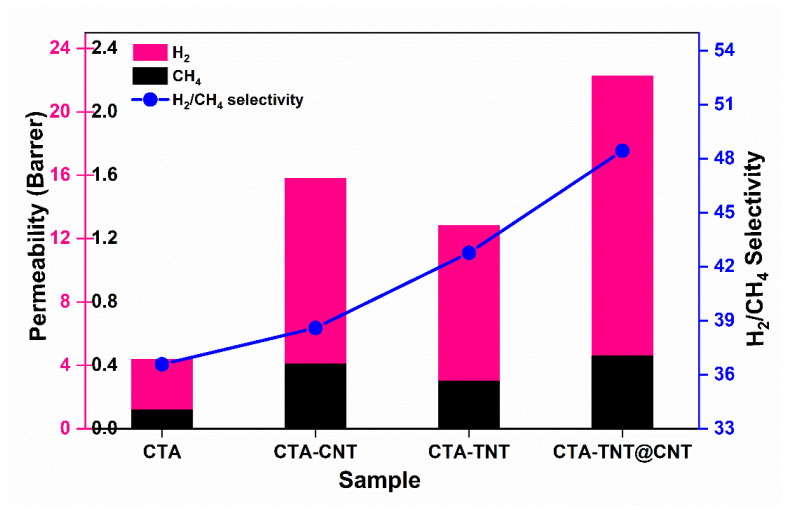
Single gas H_2_ and CH_4_ permeability and selectivity plot of the synthesized membranes.

Similarly, the H_2_/CH_4_ selectivity increased from the pristine CTA (36.58) to the membrane containing hybrid (TNT@CNT) fillers (48.43) (Figure 11 and Table 2). The permeability of H_2_ increased five-fold for the hybrid TNT@CNT-based membrane compared to the pristine CTA. The reason could be that the active fillers provided a well-aligned channel to permeate H_2_, providing a barrier for CH_4_ by including the hybrid fillers into the inter-chain of the CTA matrix. The good dispersion and adhesion properties of the CNT promoted the hybrid fillers to be homogeneously dispersed in and onto the membrane (supported by SEM images) and serve as channels to transport gas molecules. The increase in CH_4_ permeability with the addition of the hybrid fillers is assumed to be the fast diffusion of the molecules adsorbed on the surface of the fillers, as CNT is even selective toward CH_4_ [94]. Similarly, the ideal H_2_/CH_4_ selectivity as a function of the permeability of H_2_ of the synthesized membranes is illustrated using a Robeson plot in Figure S6. The synthesized membranes lay below the upper bound. However, the gas separation performance in terms of permeability and selectivity was found to be approaching toward the upper bound with the incorporation of hybrid fillers.

In general, the main gas transport pathways through the MMMs were through the dense layer of the CTA matrix, highly selective porous (TNT and CNT) fillers, and non-selective gaps or voids between the matrix and sieve particles. However, the intrinsic gas transport properties of TNT@CNT are yet unknown. The gas transport in the pristine CTA membrane followed the solution–diffusion mechanism. At the same time, the simultaneous incorporation of two different fillers with different surface chemistries and properties in the same polymer matrix is believed to provide distinct gas transport behavior. We presuppose two different diffusional paths in the process, namely, the Knudsen diffusion and solution diffusion mechanisms. Knudsen diffusion exists in the transport process through tubular fillers [95,96] aligned vertically, while solution diffusion occurs via the polymer matrixes, which is further assisted by the hybrid fillers possessing polar groups on their surface. It can also be presumed that the addition of fillers causes a tortuous path for the diffusion of larger molecules such as CH_4_ across the membrane. In contrast, such a path might not be sufficient to hinder the diffusion of smaller gas molecules such as CO_2_, thus increasing the separation efficiency. Moreover, these fillers inside the CTA polymer matrix are also presumed to possess a molecular sieving effect and form a permeable channel, allowing better gas separation than pristine membranes [12]. The observed continuous increase in permeability and selectivity upon using single fillers to hybrid fillers suggests the synergistic effect of these fillers on the gas separation behavior. Furthermore, the gas separation performance of the reported CTA-based MMMs compared to those in this study is shown in Table 3. From the comparison, we deduce that CTA-TNT@CNT MMMs present an attractive prospect for gas separation.

## 4. Conclusions

This work elucidates the effects of the combination and compactibility of the components (CNT and TNT) in hybrid MMMs. These effects were studied from different aspects, including thermal stability, dynamic mechanical behavior, structural changes, and gas separation performance, to gain better insight into hybrid MMMs fabrication using porous fillers and further modifications. SEM characterization of the membranes confirmed the compatibility of the two phases. The TGA results showed an increase in thermal resistance for MMMs compared to pristine membranes. Similarly, the mechanical properties study showed the improved strength of the membranes. Furthermore, we observed that incorporating hybrid TNT@CNT improves the overall CO_2_/CH_4_ and H_2_/CH_4_ selectivities compared to pristine CTA or even single TNT and CNT matrixes, with an overall performance closer to the upper limit of CO_2_/CH_4_ separation. This finding seems particularly interesting from a future application point of view, since commercially available polymer matrixes and easily synthesizable fillers are used as starting materials.

## Figures and Tables

**Table 1:** Glass transition temperature and mechanical properties of the synthesized membranes.

Sample Code	Glass Transition Temperature (Tg) (°C)	Mechanical Properties (25 °C)		
		Young’s Modulus (GPa)	Elongation Strain at Break (%)	Tensile Strength (MPa)
CTA	188.5	1.24 ± 0.46	51.29 ± 3.06	31.09 ± 2.28
CTA-CNT	194.7	0.70 ± 0.19	6.19 ± 1.32	37.95 ± 3.41
CTA-TNT	198.7	0.73 ± 0.30	5.28 ± 0.21	36.94 ± 3.24
CTA-CNT@TNT	198.1	1.20 ± 0.22	15.32 ± 1.32	41.21 ± 1.01

**Table 2:** Gas permeability and selectivity of the synthesized membranes.

Samples	Permeability (Barrer)			Selectivity (-)	
	H_2_	CO_2_	CH_4_	H_2_/CH_4_	CO_2_/CH_4_
CTA	4.39	3.01	0.12	36.58	25.08
CTA-TNT	12.83	8.24	0.30	42.77	27.47
CTA-CNT	15.83	14.03	0.41	38.61	34.22
CTA-TNT@CNT	22.28	19.77	0.46	48.43	42.98

**Table 3:** Gas sorption performance of the reported cellulose acetate-based MMMs in comparison to this study.

Membrane Type	Permeability (Barrer) /Selectivity (--)	References
Cellulose-based poly-ionic liquid membranes P[CA][Tf2N]	P_CO2_ = 8.9 CO_2_/CH_4_ = 22.3	Nikolaeva et al. [97]
Poly(butylene succinate)–cellulose triacetate blends [CTA + 10wt% PBS]	P_CO2_ = 3.5 CO_2_/CH_4_ = 35.0	Cihal et al. [98]
CeO_2_@GO blended CTA membrane	P_CO2_ = 10.14 CO_2_/CH_4_ = 50.7	Regmi et al. [66]
CTA/CDA blend membrane by amine functionalized ZIF-8 [CTA/CDA-NH_2_-ZIF-8 (15 wt%)]	P_CO2_ = 11.33 CO_2_/CH_4_ = 33	Raza et al. [99]
CTA and CDA blended membrane [CTA (80 wt.%): CDA(20 wt.%)	P_CO2_ = 17.32 CO_2_/CH_4_ = 18.55	Raza et al. [100]
CTA doped with ionic liquids of [emim][BF4] and [emim][dca]	P_CO2_ = 7.3 CO_2_/CH_4_ = 26	Lam et al. [54]
PEG and ZIF-8 tailored CTA membranes [CTA/PEG/ZIF-8 (60/20/20 wt.%)]	P_He_ = 73.25 He/CH_4_ = 40	Soleimany et al. [101]
APDEMS modified NaY zeolite blended CA membranes [CA/NaY-sm 20 wt.%]	P_CO2_ = 4.04 CO_2_/N_2_ = 26	Sanaeepur et al. [102]
Hybrid TNT@CNT blended CTA membrane	P_CO2_ = 19.77 CO_2_/CH_4_ = 42.98	This work

## Data Availability

Not applicable.

## Supplementary Materials

The following are available online at https://www.mdpi.com/article/10.3390/membranes11110862/s1, Figure S1. SEM images of: (A) CNT and (B) TNT nanoparticles; Figure S2. SEM and EDS overlayed images of TNT@CNT hybrid fillers; Figure S3. TEM images of: (A) TNT and (B) CNT nanoparticles; Figure S4. Image of the nature of the drop onto the membrane during the contact angle measurement of CTA and CTA-TNT@CNT MMMs; Figure S5. XRD spectra of the synthesized MMMs with different fillers; Figure S6. Plot of the H_2_/CH_4_ separation efficiency of the synthesized membranes compared to the Robeson.
