# Supplementary material for: CO2/CH4 and H2/CH4 Gas Separation Performance of CTA-TNT@CNT Hybrid Mixed Matrix Membranes
Source: Membranes (Basel). 2021 Nov 9;11(11):862. doi: 10.3390/membranes11110862 (PMC8625587; doi:10.3390/membranes11110862)
Supplement: Supplementary file 1 [file membranes-11-00862-s001.zip › membranes-1431604-supplementary.pdf]

Supplementary Information

CO_2/CH_4 and H_2/CH_4 gas separation performance of CTA-TNT@CNT hybrid mixed matrix membranes

Chhabilal Regmi[1,*], Saeed Jamali Ashtiani[1], Zdeněk Hrdlička[2], Karel Friess[1,*]

[1]Department of Physical Chemistry, University of Chemistry and Technology, Technická 5, 16628, Prague 6, Czech Republic

[2]Department of Polymers, University of Chemistry and Technology, Technická 5, 16628, Prague 6, Czech Republic

Correspondence: *regmic@vscht.cz; friessk@vscht.cz*

Results

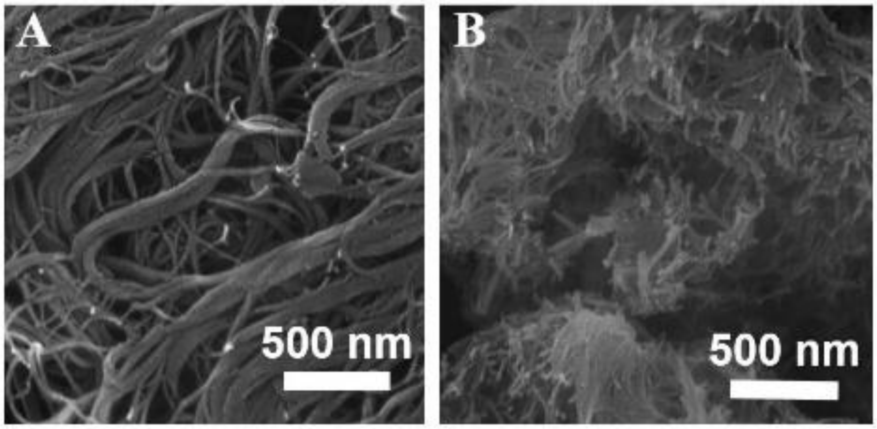

Figure S1 SEM images of; A) CNT and B) TNT nanoparticles.

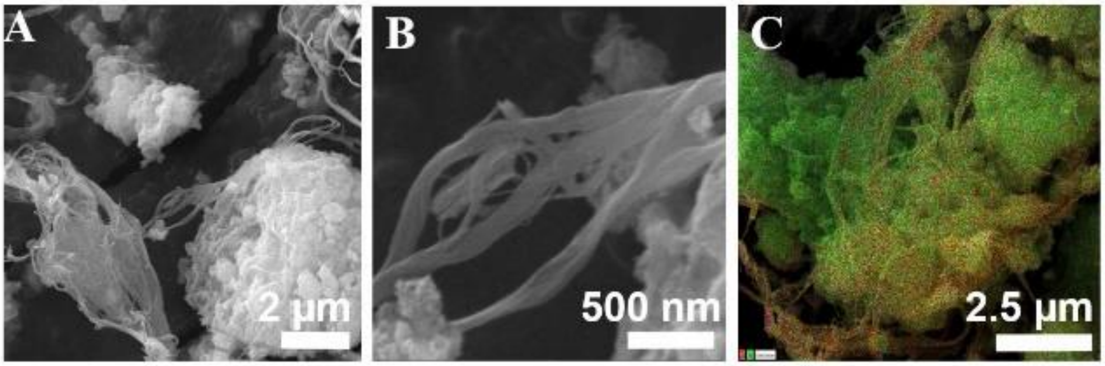

Figure S2 SEM and EDS overlayed images of TNT@CNT hybrid fillers.

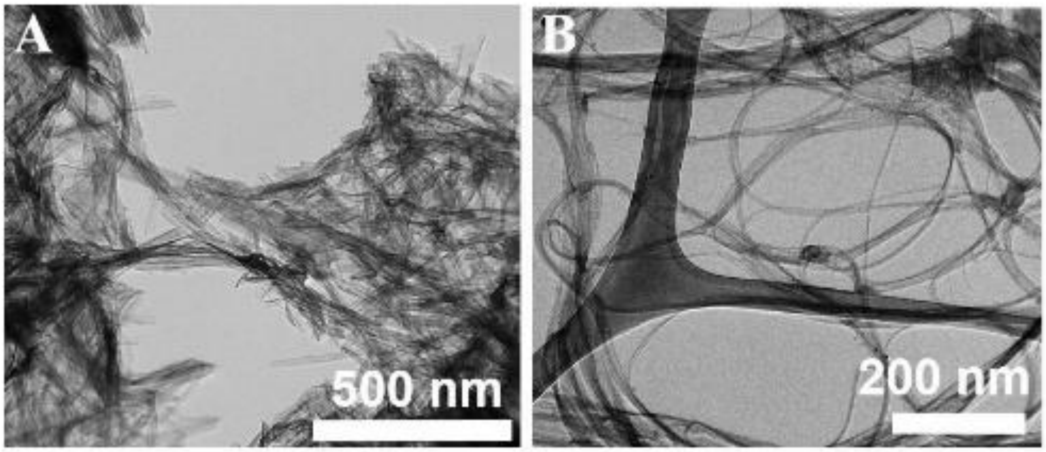

Figure S3 TEM images of; A) TNT and B) CNT nanoparticles.

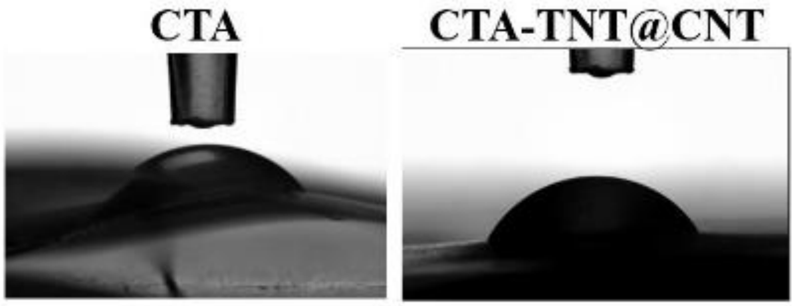

Figure S4 Image of the nature of the drop onto the membrane during contact angle measurement of CTA and CTA-TNT@CNT MMMs

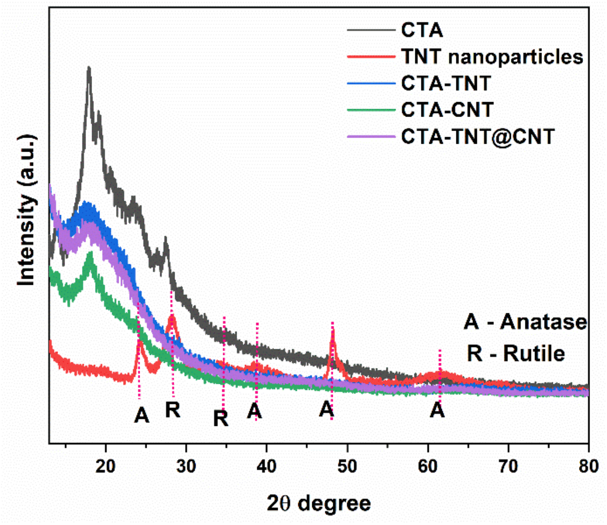

Figure S5 XRD spectra of the synthesized MMMs with different fillers.

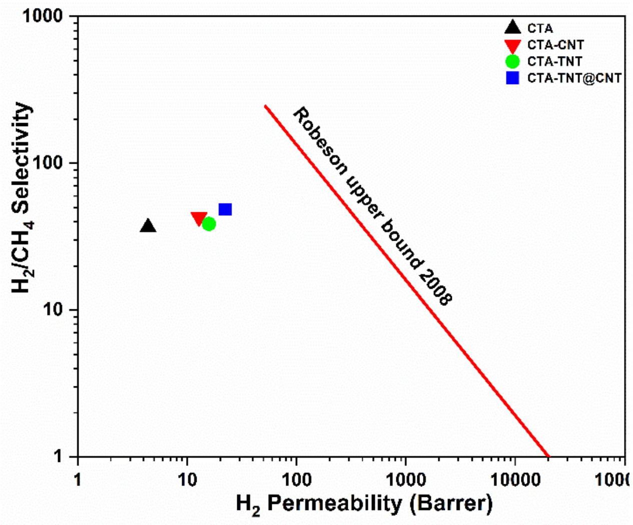

Figure S6 Plot of H_2/CH_4 separation efficiency of synthesized membranes compared with the Robeson upper-bound plot.
